# Microvesicles Contribute to the Bystander Effect of DNA Damage

**DOI:** 10.3390/ijms18040788

**Published:** 2017-04-07

**Authors:** Xiaozeng Lin, Fengxiang Wei, Pierre Major, Khalid Al-Nedawi, Hassan A. Al Saleh, Damu Tang

**Affiliations:** 1 Division of Nephrology, Department of Medicine, McMaster University, Hamilton, ON L8N 4A6, Canada; linx36@mcmaster.ca (X.L.); alnedaw@mcmaster.ca (K.A.-N.); alsaleha@mcmaster.ca (H.A.A.S.); 2 Father Sean O’Sullivan Research Institute, Hamilton, ON L8N 4A6, Canada; 3 The Hamilton Center for Kidney Research, St. Joseph’s Hospital, Hamilton, ON L8N 4A6, Canada; 4 The Genetics Laboratory, Longgang District Maternity and Child Healthcare Hospital, Longgang District, Shenzhen 518116, Guangdong, China; haowei727499@163.com; 5 Department of Oncology, McMaster University, Hamilton, ON L8V 5C2, Canada; pierrepaulmajor@gmail.com

**Keywords:** DNA damage response, microvesicles, γH2AX, ATM, ATR

## Abstract

Genotoxic treatments elicit DNA damage response (DDR) not only in cells that are directly exposed but also in cells that are not in the field of treatment (bystander cells), a phenomenon that is commonly referred to as the bystander effect (BE). However, mechanisms underlying the BE remain elusive. We report here that etoposide and ultraviolet (UV) exposure stimulate the production of microvesicles (MVs) in DU145 prostate cancer cells. MVs isolated from UV-treated DU145 and A431 epidermoid carcinoma cells as well as etoposide-treated DU145 cells induced phosphorylation of ataxia-telangiectasia mutated (ATM) at serine 1981 (indicative of ATM activation) and phosphorylation of histone H2AX at serine 139 (γH2AX) in naïve DU145 cells. Importantly, neutralization of MVs derived from UV-treated cells with annexin V significantly reduced the MV-associated BE activities. Etoposide and UV are known to induce DDR primarily through the ATM and ATM- and Rad3-related (ATR) pathways, respectively. In this regard, MV is likely a common source for the DNA damage-induced bystander effect. However, pre-treatment of DU145 naïve cells with an ATM (KU55933) inhibitor does not affect the BE elicited by MVs isolated from etoposide-treated cells, indicating that the BE is induced upstream of ATM actions. Taken together, we provide evidence supporting that MVs are a source of the DNA damage-induced bystander effect.

## 1. Introduction

The World Health Organization has thoroughly documented that radiation induces cancer [1]. Major epidemiological studies on A-bomb survivors clearly linked radiation exposure to carcinogenesis for human cancers [2]. Mechanistically, radiation causes DNA damage when traveling through the nucleus of the cell, thereby inducing DNA damage response (DDR) by activating three apical PI3 kinase related kinases (PIKKs): ATM (ataxia-telangiectasia mutated), ATR (ATM- and Rad3-related), and DNA-dependent protein kinase (DNAPK) [3,4,5]. ATM and ATR subsequently phosphorylate their downstream targets, including check kinase 1 (CHK1) S345 (for ATR), CHK2 T68 (for ATM), and H2AX S139 (γH2AX) [5,6]. The actions of ATM, ATR and DNAPK orchestrate check point activation and DNA lesion repair [3,4,5].

As a result of being exposed to genotoxic treatment, cells not only elicit DDR but also produce factors that induce DDR in cells that have not been exposed to genotoxic reagents, a process that is commonly known as the bystander effect (BE) of DNA damage [7,8]. Bystander factors released from irradiated cells play critical roles in radiotherapy-associated secondary carcinogenesis [9]. These secondary cancers were observed in patients with cervical and breast cancer [10,11,12,13,14,15]; the reported latency is 5–15 years for cervical cancer patients receiving external bean radiation [16]. In a 7-year follow-up of 269,069 prostate cancer patients treated with radiotherapy, approximately 10% experienced secondary cancers [16,17]. It has been shown that the blood of patients receiving radiotherapy contained activities or clastogenic factors that cause chromosome damage [18,19,20]. Clastogenic factors were also detected in the plasma of individuals exposed to radiation from the Chernobyl nuclear reactor accident [21]. Additionally, conditioned medium harvested from irradiated cells induced cell death, mutation, and genome instability in naive cells [22,23,24,25,26,27,28], supporting the concept that the clastogenic factors detected in the plasma of radiotherapy-treated patients were directly released from irradiated cells. Furthermore, local cranial irradiation induced distance BE DNA damage in mice in the lead-shield spleen and testes [29,30] and irradiation of one side of the mouse body caused DNA damage and epigenetic changes in the other side of the body that was shielded by lead [31]. More importantly, irradiation of the lower body induced medulloblastoma in the brain of Patched 1 heterozygous mice [32].

While accumulating evidence demonstrates that bystander DNA damage contributes to radiotherapy-induced secondary cancers, the bystander factors remain unclear. Although a variety of molecules have been implicated in mediating the BE, including reactive oxygen species (ROS), reactive nitrogen species, TNFα, TGFβ1, IL-6, and IL-8 [9], evidence suggests the existence of additional bystander factors [7].

Microvesicles (MVs) are small membrane-enclosed sacks that are shed from donor cells. MVs carry a unique set of cargo, and communicate the specific messages from donor cells to the acceptor cells [33,34,35]. As bystander factors communicate the stress signals originated from cells undergoing DNA damage response to naïve cells, MVs are ideal vesicles to carry these messages to bystander cells. We thus report here that MV is a source of bystander DNA damage signals.

## 2. Results

### 2.1. DNA Damage Enhances Microvesicles (MV) Formation

**Figure 1:**
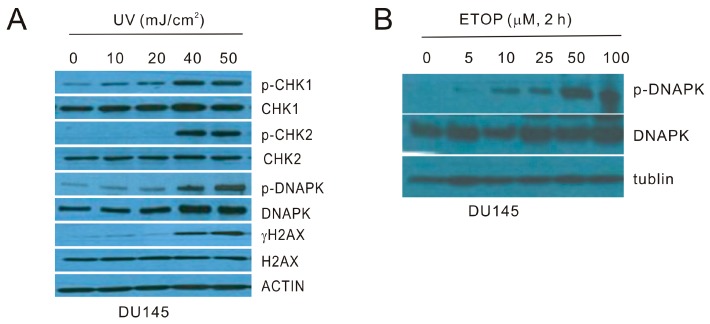
Characterization of ultraviolet (UV) and etoposide (ETOP) induced DNA damage response (DDR). (**A**) DU145 cells were treated with UV at the indicated energy levels and cultured for 6 h. Western blot was performed for the indicated proteins. p-DNAPK: phosphorylation of DNAPK at serine 2056 (S2056); p-CHK1: phosphorylation of CHK1 at S345; p-CHK2: phosphorylation of CHK2 at threonine 68 (T68); (**B**) DU145 cells were treated with ETOP for 2 h at the indicated doses, followed by Western blot examination for the indicated proteins. All experiments were repeated once; typical images from a single repeat are shown.

ATM and ATR play pivotal roles in the initiation of DDR under different settings of DNA damage. While etoposide (ETOP) primarily activates ATM by the induction of double strand DNA breaks (DSBs) [3,36,37], ultraviolet (UV)-initiated DDR is mediated by ATR [38,39]. We thus set up our investigations into the involvement of microvesicles (MVs) in ETOP and UV-induced BE. For this purpose, we first determined the kinetics of UV and etoposide-elicited DDR in DU145 cells. In response to UV at the energy levels ranging from 10 to 50 mJ/cm^2^, CHK1 phosphorylation at S345 (p-CHK1) was clearly induced in a dose-dependent manner starting at 10 mJ/cm^2^ in DU145 cells, while other typical DDR events, including CHK2 phosphorylation at T68 (p-CHK2), phosphorylation of DNAPKcs (catalytic subunit) at S2056 (p-DNAPK), and γH2AX production, occurred in response to treatment with higher doses (Figure 1A). ATR plays a major role in the initiation of DDR caused by UV exposure [40]; CHK1 is a specific ATR target [41,42,43,44]. The kinetics of UV-induced DDR events in DU145 cells are thus in line with this knowledge. On the other hand, etopside results in DSBs, which primarily induce ATM and DNAPK activation [41,45,46]. In this regard, ETOP induces DNAPK activation, evidenced by phosphorylation of DNAPK at S2056, in a dose-dependent manner in DU145 cells (Figure 1B).

**Figure 2:**
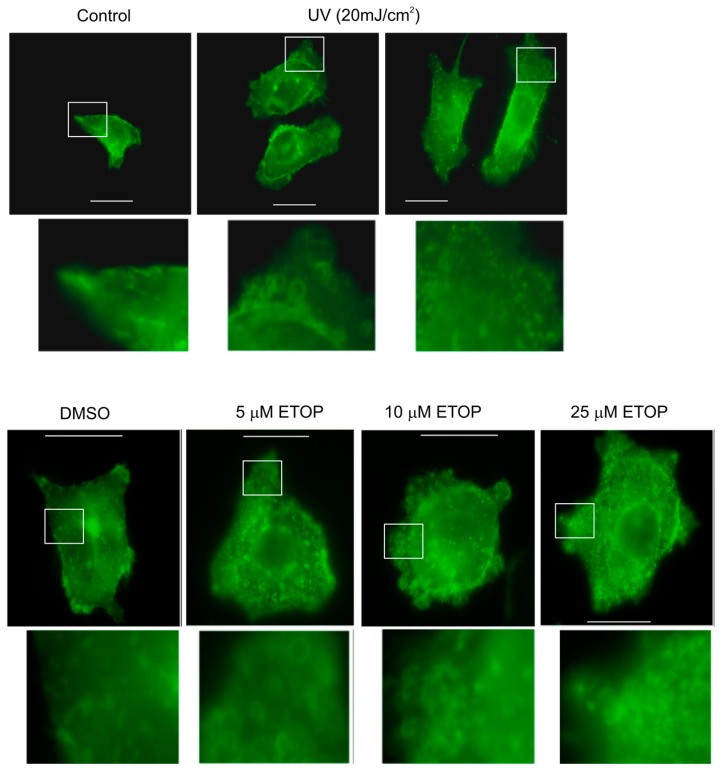
UV and ETOP treatments elevate microvesicle production. DU145 cells were stably expressed with a membrane-bound green fluorescent protein (GFP). The cells were treated with UV at 20 mJ/cm^2^ and cultured for 6 h or with the indicated doses of ETOP for 2 h. Images were taken for control treatment, UV-, DMSO- and ETOP-treated cells. Scale bars indicate 20 μm. The marked regions are enlarged 4-fold and placed underneath the respective panels. Experiments were repeated once; typical images are included.

We subsequently analyzed the effects of UV and ETOP treatment on MV production. It has been reported previously that expression of a membrane-bound green fluorescent protein (GFP) simplified the process of detecting MVs [47]. Following this system, we have stably expressed membrane-bound GFP in DU145 cells. In comparison to mock-treated cells, UV at 20 mJ/cm^2^ and ETOP at concentrations ranging from 5 μM to 25 μM increased the number of MVs per cell (Figure 2). Our results are consistent with a report showing an elevation of MV production in MCF7 cells undergoing doxorubicin-induced DDR [48]. Etoposide and doxorubicin are well-established DNA topoisomerase II inhibitors [49].

### 2.2. MVs Contributes to DNA Damage-Induced Bystander Effect (BE)

**Figure 3:**
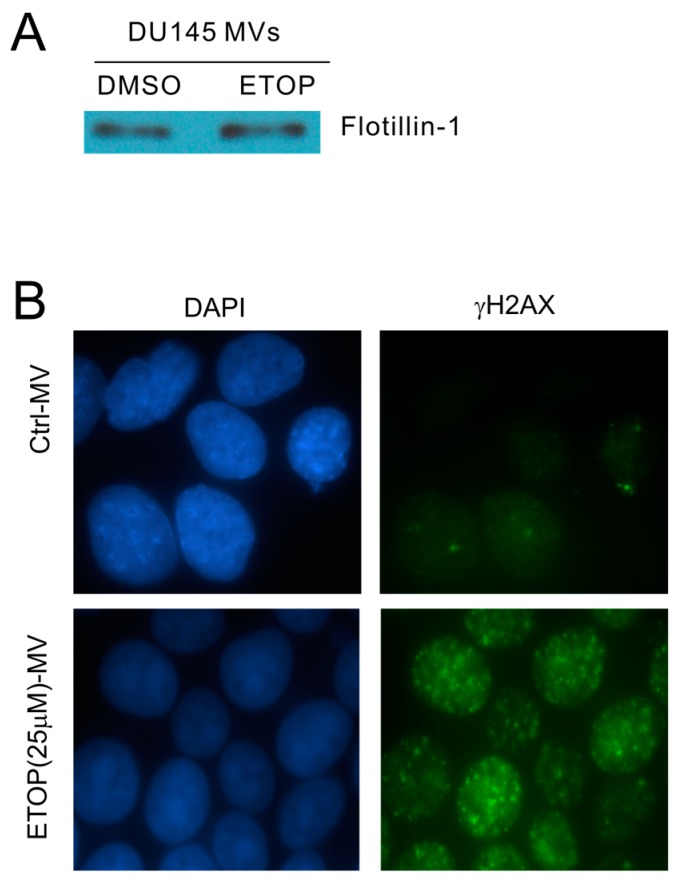
Microvesicles (MVs) derived from ETOP-treated DU145 cells induce the bystander effect (BE) in naïve DU145 cells. (**A**) MVs were isolated from DU145 cells treated with DMSO (control/Ctrl) or 25 μM ETOP for 24 h, followed by Western blot for flotillin-1; (**B**) DU145 recipient (naïve) cells were incubated with Ctrl-MVs or ETOP-MVs at 100 μg/mL for 24 h, followed by immunofluorescence (IF) staining for γH2AX. Nuclei were counterstained with DAPI. Images were acquired at 63× magnification.

The observed enhancement of MV production in cells undergoing DDR above strongly suggests that MVs produced by these cells are able to elicit DDR in naïve or bystander cells. To examine this possibility, we isolated MVs from DU145 cells treated with either DMSO or 25 μM ETOP; these isolations were confirmed by the presence of flotillin (Figure 3A), a well-established MV protein [50]. At 100 μg/mL, MVs derived from ETOP-treated cells (ETOP-MVs) robustly induced γH2AX in naïve DU145 cells in comparison to MVs produced by DMSO-treated cells (control/Ctrl-MVs) (Figure 3B). The bystander DDR was unlikely resulted from residue ETOP presence in ETOP-MVs, as similar observations were reported when naïve MCF7 cells were incubated with MVs isolated from doxorubicin-treated cells [48].

**Figure 4:**
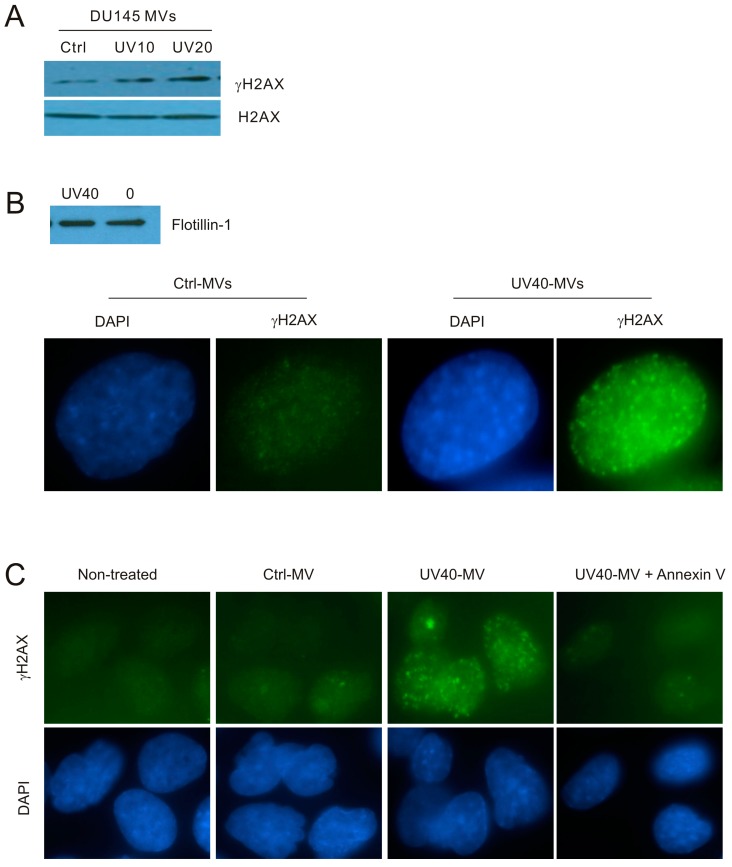
MVs produced by UV-treated DU145 cells induce the BE in recipient DU145 cells. (**A**) MVs were isolated from DU145 cells treated with Ctrl or UV at either 10 mJ/cm^2^ (UV10) or 20 mJ/cm^2^ (UV20) and used to incubate with naïve DU145 cells at 100 μg/mL for 24 h, followed by Western blot examination for γH2AX and H2AX. Experiments were repeated once; typical images from a single repeat are included; (**B**) MVs were isolated from control (0) or UV-treated (40 mJ/cm^2^; UV40) DU145 cells; MV isolations were confirmed by Western blot for flotillin (**top** panel). DU145 naïve cells were incubated with Ctrl-MVs and UV40-MVs for 24 h, followed by IF examination for γH2AX. Nuclei were counterstained with DAPI. Typical images are shown (**bottom** panel); Images were taken at the 100× magnification. (**C**) DU145 naïve cells were either untreated (non-treated) or treated with Ctrl-MVs, UV40-MVs, or UV40-MVs + annexin V. IF was carried out for γH2AX. Nuclei were counterstained with DAPI. Images were acquired at the 63× magnification.

To further study the contributions of MVs to the DNA damage-induced BE in naïve cells, we have treated DU145 cells with UV at either 10 or 20 mJ/cm^2^, conditions that resulted in DDR evidenced by elevations of p-CHK1 (Figure 1A). In comparison to MVs isolated from control (Ctrl) cells, MVs derived from cells treated with UV at either 10 mJ/cm^2^ (UV10) or 20 mJ/cm^2^ (UV20) clearly enhanced γH2AX production in DU145 naïve cells (Figure 4A). Additionally, MVs derived from UV-treated DU145 cells induced γH2AX nuclear foci in recipient DU145 cells in comparison to Ctrl MVs (Figure 4B). These observations further exclude the unlikely possibility that MVs produced from ETOP-treated DU145 initiate the BE through a potential presence of residue ETOP. Furthermore, by taking advantage of the high affinity association between annexin V and MVs [50,51], we were able to demonstrate that incubation of MVs isolated from DU145 cells treated with UV at 40 mJ/cm^2^ with annexin V effectively blocked the MV-associated BE activities (Figure 4C).

**Figure 5:**
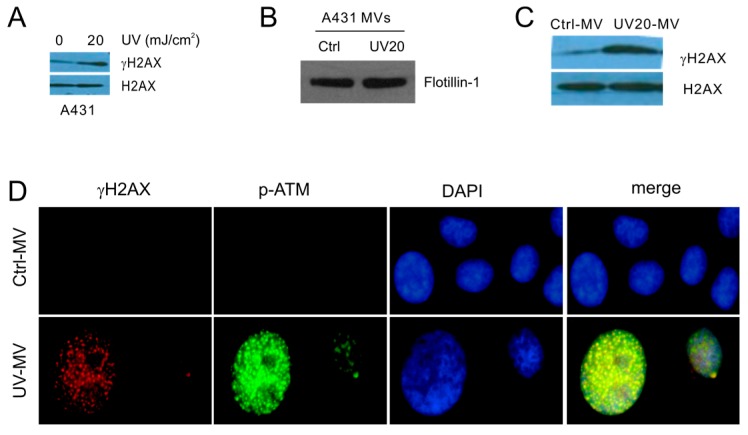
MVs produced by UV-treated A431 cells induce the BE in naive DU145 cells; (**A**) A431 cells were control- or UV-treated as indicated, cultured for 6 h, and examined for γH2AX and H2AX; (**B**) A431 cells were treated with Ctrl or UV at 20 mJ/cm^2^ (UV20) and cultured for 6 h. Isolation of MVs was examined for flotillin by Western blot; (**C**,**D**) DU145 recipient cells were treated with Ctrl- or UV20-MVs for 24 h at 100 μg/mL, followed by Western blot examination for γH2AX and H2AX (**C**) and by IF for γH2AX or S1981-phosphorylated ATM (p-ATM). Nuclei were counterstained with DAPI. Images were taken at the 63× magnification (**D**).

The bystander DDR properties can also be demonstrated in heterogeneous cell types. We first established that UV at 20 mJ/cm^2^ induced DDR in A431 cells (Figure 5A). Following these conditions, we initiated DDR in A431 cells through UV exposure, and subsequently isolated MVs from Ctrl and UV-treated A431 cells (Figure 5B). In comparison to MVs isolated from Ctrl A431 cells, those derived from UV-treated A431 cells enhanced γH2AX production in recipient DU145 cells (Figure 5C). Furthermore, MVs produced by UV-treated A431 cells clearly generated γH2AX and p-ATM nuclear foci, indicative of ATM activation, in DU145 naïve cells (Figure 5D); co-localization of both types of nuclear foci was also demonstrated (Figure 5D). Taken together, we provide evidence supporting that MVs produced from DDR cells possess activities in inducing BE.

### 2.3. MV Induces BE in Recipient Cells Upstream of Ataxia-Telangiectasia Mutated (ATM)

**Figure 6:**
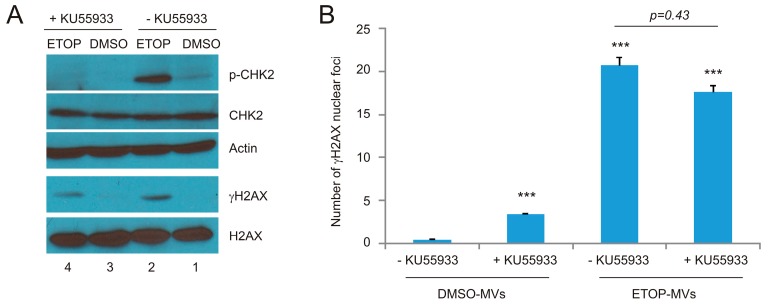
Inhibition of ATM activities in recipient DU145 cells does not reduce the BE induced by MVs. (**A**) DU145 cells were pre-treated with the ATM inhibitor KU55933, followed by mock treatment with DMSO or ETOP at 25 μM for 2 h. Western blot analysis was subsequently performed for the indicated proteins; (**B**) DU145 cells were pre-incubated with KU55933 for 8 h, followed by treatment with DMSO-MVs or ETOP-MVs (derived from cells treated with 25 μM ETOP for 24 h) for 24 h. IF staining was carried out for γH2AX. Experiments were repeated three times. More than 400 nuclei in several randomly selected fields were counted for γH2AX nuclear foci; means ± SE (standard error of the mean) were graphed. *** *p* < 0.0001 in comparison to naïve DU145 cells treated with DMSO MVs (2-tailed Student *t*-test).

In view of the respective apical role of ATM and ATR in ETOP and UV-initiated DDR [36,37,40], we have investigated the respective contributions of ATM and ATR in the ETOP- and UV-MV-induced BE. For this purpose, we first demonstrated that the ATM inhibitor KU55933 at 10 μM substantially reduced ETOP-induced CHK2 phosphorylation, a well-established ATM target (Figure 6A). Furthermore, the basal level of CHK2 phosphorylation was also inhibited by KU55933 (Figure 6A, comparing p-CHK2 in lane 1 and lane 3). On the other hand, KU55933 does not apparently affect ETOP-induced γH2AX production (Figure 6A), consistent with the fact that γH2AX is also produced by ATR and DNAPK [5,6]. Collectively, evidence supports that KU55933 at 10 μM inhibits ETOP-initiated ATM activation. Consistent with a major role of ATM activity in DSB repair [4,52], the presence of KU55933 sensitized γH2AX formation in DU145 cells treated with DMSO MVs (Figure 6B). However, KU55933 did not significantly affect γH2AX nuclear foci in naïve DU145 cells treated with ETOP-MVs (Figure 6B). These results do not support a major role of ATM kinase activity in the ETOP MV-induced BE.

**Figure 7:**
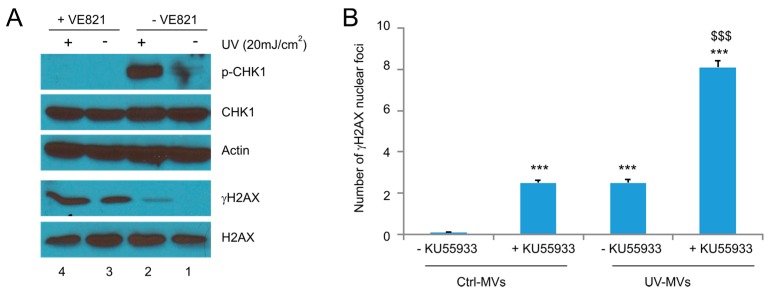
Examination of the involvement of ATR activities in naive DU145 cells treated with MVs derived from UV-treated DU145 cells. (**A**) DU145 cells were pre-treated with the ATR inhibitor VE821 for 8 h, followed by exposure to UV at 20 mJ/cm^2^ and cultured for 6 h. Western blot was then performed for the indicated events; (**B**) DU145 naïve cells were pre-incubated with VE821 for 8 h, followed by the treatment of Ctrl-MVs or UV-MVs (derived from DU145 cells treated with UV at 20 mJ/cm^2^) for 24 h. IF staining was carried out for γH2AX. Experiments were repeated three times. More than 500 nuclei in several randomly selected fields were counted for γH2AX nuclear foci; means ± SE (standard error of the mean) were graphed. *** *p* < 0.0001 in comparison to naïve DU145 cells treated with Ctrl-MVs (2-tailed Student *t*-test); ^$$$^ *p* < 0.0001 in comparison to naïve DU145 cells treated with UV-MVs (2-tailed Student *t*-test).

Following the same principle, we have examined the involvement of ATR in the UV-MVs-induced BE. By taking advantage of CHK1 being the most specific ATR target in DDR, we were able to show that both the basal and UV-induced p-CHK1 were dramatically reduced by the specific ATR inhibitor VE821 (Figure 7A). Surprisingly, VE821 significantly elevated γH2AX production (Figure 7A, comparing lane 1 to lane 3) and γH2AX nuclear foci in DU145 recipient cells treated with not only Ctrl-MVs but also UV-MVs (Figure 7B). The enhancement was likely attributable to ATR activity being required in preventing the collapse of replication forks during DNA replication; inhibition of ATR activity by VE821 itself will be expected to induce DDR. In this regard, the system was unable to examine the role of ATR in the UV-MV-elicited BE.

## 3. Discussion

A large body of evidence reveals a major impact of the DNA damage-induced BE on human health. Radiotherapy is a major cancer therapy and can effectively control tumors. However, the treatment is associated with a variety of chronic complications, including the well-established cardiovascular diseases [53], and secondary malignancies. These adverse effects occur in both adult and childhood cancer survivors, and are a particular concern for the latter survivors. While technological advances in modern radiotherapy enable 80% of children with cancer to survive for 5 years or longer [54,55], the cumulative incidence of secondary neoplasm was 20.5% among these survivors 30 years later [54,56]. Additionally, chronic health conditions affect 73.4% of survivors with 42.4% being life-threatening 30 years after radiotherapy [57]. Despite the BE being a major health issue in cancer patients receiving radiation therapy, the process has not been thoroughly investigated and the underlying mechanisms remain elusive.

We provide clear evidence supporting a role of MVs in inducing the BE. This concept is based on our observations that MVs derived from cells undergoing DDR induce the BE in naïve DU145 cells and that neutralization of these MVs with annexin V significantly reduces the BE activities. The concept of MVs as a DDR messenger is supported by the knowledge that MVs play roles in cell-to-cell communication [34,58,59]. Intriguingly, MVs are a pathological contributor to cardiovascular disease [58], a well-established adverse effect caused by radiation therapy in cancer patients [53]. Furthermore, the involvement of MVs in the DDR-induced BE has also been reported by others very recently [48,60].

In line with our observations, Carroll et al. reported an elevation of MV shedding in doxorubicin-treated MCF7 cells [48]. In addition to etoposide, we also observed an enhancement in MV shedding in DU145 cells treated with UV (Figure 2). Based on these observations, it is tempting to propose that upregulation of MV shedding is a general response of cells undergoing DDR and that this upregulation has a functional consequence i.e., the bystander effect.

MVs contain a complex array of materials that are able to mediate cell–cell communications [34]. It is thus likely that multiple mechanisms are in play for DDR cell-derived MVs to elicit the BE in naïve cells. ATM, BRCA1, FANCD2, and CHK1 have been indicated to contribute to the BE in naïve cells in response to conditioned medium obtained from cells treated with ionizing radiation [61,62]. Transfer of microRNA-21 (miR-21) by MVs was reported to be a cause of ionizing radiation-induced BE [60]. In our research, inhibition of ATM activation using KU55933 in recipient cells was without an apparent impact on the formation of γH2AX nuclear foci when treated with MVs produced by etoposide-treated DU145 cells (Figure 6B). The nuclear focus of γH2AX is a common surrogate marker of DSBs [63], suggesting that MVs derived from cells with DNA damage are able to damage DNA. However, the molecular basis underlying this process remains unknown. Collectively, the exact mechanisms underlying the MV-mediated BE need further investigations.

The BE is observed in recipient DU145 cells treated with MVs isolated from DU145 and A431 cells exposed to either etoposide or UV (this study). MVs also deliver the BE message in MCF7 breast cancer and human lung fibroblast MRC-5 cells in the setting of doxorubicin and ionizing radiation-induced DDR [48,60]. Taken together, these observations suggest the existence of common BE factors in DDR cell-produced MVs. However, this possibility does not exclude the presence of other BE factors in cells undergoing DDR. Nonetheless, the contributions of MVs to the DDR-induced BE implies an interesting application of annexin V in attenuating the chronic effects of radiation therapy.

## 4. Materials and Methods

### 4.1. Chemicals, Cell Lines, and Plasmids

Hydroxyurea (HU) and etoposide (ETOP) were purchased from Sigma (Oakville, ON, Canada). ETOP was dissolved in dimethylsulfoxide (DMSO). Annexin V was purchased from BD Biosciences (Mississauga, ON, Canada). DU145 and A431 cells were obtained from American Type Culture Collection (ATCC, Manassas, VA, USA), and cultured in Mininum Essential Medium Eagle (MEM) (DU145) and Dulbecco’s Modified Eagle Medium (DMEM) (A431) media supplemented with 10% foetal bovine serum (FBS; Sigma Aldrich, Oakville, ON, Canada) and 1% penicillin–streptomycin (Life Technologies; Burlington, ON, Canada). Membrane-associated GFP (green fluorescent protein) (mGFP) was provided by Addgene (Cambridge, MA, USA) and subcloned into PLPCX retroviral vector. DU145 cells stably expressing mGFP were subsequently constructed using mGFP retrovirus.

### 4.2. Retroviral Infection

Packing retrovirus was performed according to our published procedures [64,65,66]. Briefly, the mGFP retroviral vector, the gag-pol (GP) and an envelope expressing vector (VSV-G) (Stratagene, Mississauga, ON, Canada) vectors were transiently co-transfected into 293T cells using a calcium-phosphate transfection. The virus-containing medium was harvested 2 days later, filtered using a 0.45 μM filter, and centrifuged at 50,000× *g* for 90 min. The viral pellet was resuspended in the MEM medium containing 10 μg/mL of polybrene (Sigma) prior to infecting cells. Infection was selected using specific antibiotics.

### 4.3. Immunofluorescence Staining

Immunofluorescence (IF) staining was performed following our published conditions [64,65,66]. Briefly, cells were fixed with prechilled (−20 °C) acetone–methanol for 15 min prior to the addition of primary antibodies anti-γH2AX (1:100, Cell Signaling, Danvers, MA, USA) and anti-phospho-ATM (S1981) (1:100, Cell Signaling) at 4 °C overnight. After rinsing, FITC-Donkey anti-mouse IgG (1:200, Jackson Immuno Research Lab, West Grove, PA, USA) and Rhodamine-Donkey anti-rabbit IgG (1:200, Jackson Immuno Research Lab) were added for 1 h at room temperature. Slides were mounted using the VECTASHIELD mounting medium containing DAPI (VECTOR Lab Inc., Burlington, ON, Canada). VE-281 (Selleckchem, Burlington, ON, Canada) or KU-55933 (Selleckchem) was added to cells 8 h prior to treatment. Images were then acquired with a fluorescent microscope (Axiovert 200, Carl Zeiss, North York, ON, Canada).

### 4.4. Western Blot Analysis

Cells were lysed in a buffer containing Tris (20 mM, pH 7.4), NaCl (150 mM), EDTA (1 mM), EGTA (1 mM), Triton X-100 (1%), sodium pyrophosphate (25 mM), NaF (1 mM), β-glycerophosphate (1 mM), sodium orthovanadate (0.1 mM), PMSF (1 mM), leupeptin (2 μg/mL) and aprotinin (10 μg/mL). An amount of 50 μg of total cell lysate protein was separated on SDS-PAGE gel, and transferred onto Hybond ECL nitrocellulose membranes (Amersham, UK), which were blocked with 5% skim milk at room temperature (RT) for 1 h, and incubated with individual primary (overnight at 4 °C) and secondary antibodies for 1 h at RT. Signals were subsequently developed using an ECL kit (Amersham, UK). Primary antibodies used were anti-γH2AX (1:100, Cell Signaling); anti-H2AX (1:1000, Millpore, Billerica, MA, USA); anti-phosphorylated DNAPK (1:1000, Abcam, Toronto, ON, Canada); anti-DNAPK (1:1000, Abcam); anti-phosph-CHK1 (S345) (1:500, Cell Signaling); anti-CHK1 (1:1000, Cell Signaling); anti-phospho-CHK2 (T68) (1:1000, Cell Signaling); anti-CHK2 (1:1000, Cell Signaling); anti-Flotillin-1 (1:1000, Cell Signaling); and anti-actin (1:1000, Santa Cruz, Dallas, TX, USA).

### 4.5. Microvesicle Isolation and Treatment of Cells with MVs

Isolation of microvesicles was carried out based on our published procedures [50,51,67]. Briefly, conditioned medium was harvested from cells following specific treatments, followed by centrifugations for 5 min at 300× *g*, 20 min at 12,000× *g*, and 120 min at 100,000× *g.* Supernatant of the first two centrifugations and the pellet of the last centrifugation were collected. The 100,000× *g* centrifugation pellet was washed twice with phosphate buffered saline (PBS), and resuspended in PBS. The exosome proteins were determined using the Bradford assay (Bio-Rad, Mississauga, ON, Canada). Cells were treated with MVs at 100 μg/μL and MVs that were pre-incubated with Annexin V (BD Pharmingen, Mississauga, ON, Canada) at 2 μg/mL for 1 h.

### 4.6. Statistical Analysis

Student’s *t*-test (2-tails) was used for statistical analyses. The significant level was defined as a *p*-value < 0.05.
